# Immunosuppression in Early Postnatal Days Induces Persistent and Allergen-Specific Immune Tolerance to Asthma in Adult Mice

**DOI:** 10.1371/journal.pone.0122990

**Published:** 2015-04-10

**Authors:** Yan Chen, Jin Zhang, Yong Lu, Libo Wang

**Affiliations:** Department of Respiratory Medicine, Children’s Hospital of Fudan University, Shanghai, China; Research Center Borstel, GERMANY

## Abstract

Bronchial asthma is a chronic airway inflammatory condition with high morbidity, and effective treatments for asthma are limited. Allergen-specific immunotherapy can only induce peripheral immune tolerance and is not sustainable. Exploring new therapeutic strategies is of great clinical importance. Recombinant adenovirus (rAdV) was used as a vector to make cells expressing cytotoxic T lymphocyte-associated antigen-4-immunoglobulin (CTLA4Ig) a soluble CTLA4 immunoglobulin fusion protein. Dendritic cells (DCs) were modified using the rAdVs together with allergens. Then these modified DCs were transplanted to mice before allergen sensitization. The persistence and specificity of immune tolerance were evaluated in mice challenged with asthma allergens at 3 and 7 months. DCs modified by CTLA4Ig showed increased IL-10 secretion, decreased IL-12 secretion, and T cell stimulation in vitro. Mice treated with these DCs in the early neonatal period developed tolerance against the allergens that were used to induce asthma in the adult stage. Asthma symptoms, lung damage, airway reactivity, and inflammatory response all improved. Humoral immunity indices showed that this therapeutic strategy strongly suppressed mice immune responses and was maintained for as long as 7 months. Furthermore, allergen cross-sensitization and challenge experiments demonstrated that this immune tolerance was allergen-specific. Treatment with CTLA4Ig modified DCs in the early neonatal period, inducing persistent and allergen-specific immune tolerance to asthma in adult mice. Our results suggest that it may be possible to develop a vaccine for asthma.

## Introduction

Bronchial asthma is a chronic airway inflammatory condition involving mainly eosinophils but with contributions from many other cell types and inflammatory mediators [1,2]. The current treatment of bronchial asthma depends mainly on glucocorticoids, although they only treat symptoms and do not improve the prognosis of asthma [2]. The increasing incidence of asthma in children is highly correlated with allergens, and the limitations of glucocorticoid treatment make it important to develop new therapeutic strategies to change the allergic inflammatory process [2, 3].

Allergen-specific immunotherapy (ASIT) is the only treatment that affects the natural progress of allergic diseases [4,5]. The principle of ASIT is to induce and maintain allergen-specific T cell peripheral immune tolerance [4–6]. Immune tolerance is the specific immune suppression of a particular antigen [7]. This immunological unresponsiveness is only to the specific antigen in question and does not affect the overall function of adaptive immune responses generally [8]. During the development process, T and B lymphocyte clone responses to a specific antigen could be tolerated with immune tolerance induction [8, 9]. This immune tolerance will recede gradually when the induction is eliminated. This is referred to as peripheral immune tolerance [4–10]. Current clinical ASIT is based mainly on this theory [4–10].

In the embryonic stage, immature T and B lymphocytes that encounter foreign antigens will always form immune tolerance to those antigens after birth [11, 12]. In principle, this tolerance will continue for life and is usually called central tolerance [13]. Central tolerance is the mechanism by which newly developing T and B cells are rendered non-reactive to self, which is achieved when thymocytes with high affinity for self-peptides/major histocompatibility complex undergo negative selection [14, 15].

Studies have shown that the central tolerance mechanism is still in progress after birth [16]. Defects in the postnatal thymus and bone marrow stromal cells and negative selection disorders will increase the incidence of autoimmune diseases [17, 18]. If mice of strain H-2a are transplanted with bone marrow from CBA (H-2k) strain mice in the neonatal period, and then donor skin is transplanted to 8-week-old H-2a strain mice, the skin graft can survive for a long time without immune rejection [18]. This experiment suggested that the newborn period is a good stage for intervention regarding immune tolerance. Intervention in this phase can lead to central tolerance, and this tolerance will be maintained for life, at least in principle. Thus, the central tolerance mechanism provides a possible solution to improve ASIT treatments for bronchial asthma.

Dendritic cells (DCs) as professional antigen-presenting cells, are the initial cells of asthma and other types of allergic inflammation [19]. DCs can produce allergic reactions and can also induce immune tolerance [20]. Thus, intervention in the antigen-presenting process completed by DCs is a possible entry point for allergic inflammation intervention. The CD80/CD86-CD28 axis is an important pathway for immuno-corrective therapy. The CTLA4 has one log higher competitive binding activity to CD80/CD86 than CD28 and is widely used in studies of immuno-corrective therapy [21, 22]. Naïve DCs overexpressing CTLA4 from a recombinant adenovirus vector have a therapeutic effect on asthma in adult mice [23, 24]. However, this therapeutic approach depends on a peripheral immune tolerance mechanism and would be expected to gradually fade.

To improve the limitations of present peripheral tolerant therapy and guarantee the overall function of adaptive immune response, we tried to intervene in the B and T cell tolerance process during the development of newborn mice using the transplantation of CTLA4Ig-modified DCs. The effects and specificity of this immune suppression in the therapy of bronchial asthma were evaluated at the sensitized and excitation levels, in both adolescent and old mice asthma models.

## Materials and Methods

### Mice

BALB/c (H-2Kd, I-Ad) mice were maintained under specific pathogen-free (SPF) conditions at the Animal Resources Centre, Fudan University. Mice were raised on an OVA- and Derp-free diet (Sigma, St. Louis, MO, USA). All animal experiments were conducted in accordance with internationally recognized guidelines for animal experiments (Animal Research: Reporting *in vivo* Experiments guidelines) and were approved by the Animal Ethics Committee of Children’s Hospital of Fudan University (reference no. 2012–0017).

### Allergen sensitization, challenge, and treatment

In most experiments, BALB/c mice were divided into the following groups: saline-treated control group, OVA- or Derp-sensitized/challenged asthma group, OVA or Derp plus rAdV-CTLA4Ig-modified DCs transplanted, OVA- or Derp-sensitized/challenged group, and OVA or Derp plus rAdV-GFP-modified DCs transplanted, and OVA or Derp-sensitized/challenged group.

Each group contained 10 mice. In specificity tests, Derp purified from house dust mites (Biovisualab, Shanghai, China) was used to corroborate the specificity of immune tolerance.

For the allergen-induced murine model of asthma, allergen sensitization and inhalational antigen challenge were performed as follows. Briefly, 7- to 11-day-old neonatal mice were sensitized by intraperitoneal and thigh subcutaneous injection of 200 μL of a solution containing 100 μg OVA or 5 μg Derp mixed with aluminum hydroxide (0.5 mg/mL; Sigma). Three or seven months later, the sensitized mice were placed in a 14 × 11 × 11 cm^3^ plastic chamber and exposed to aerosolized phosphate- buffered saline (PBS) containing 1% OVA or Derp or PBS for 30 min. The control mice were given injections of saline and PBS aerosols, similar to the OVA-treated mice. For mice treated with DCs, prior to the first inhalational antigen challenge, 1×10^6^ rAdVs-infected DCs were administered intravenously.

### Recombinant CTLA4Ig adenovirus vectors and quality control of rAdVs

The ectodomain of the human *CTLA4* gene (NCBI Reference Sequence: NM_001037631.2) was fused to the coding sequence of IgCγ Fc to ensure CTLA4Ig secretion [23, 24]. This DNA fragment was inserted into a replication-defective recombinant adenovirus (Agilent Technologies, La Jolla, CA) to generate the CTLA4Ig viral vector rAdV-CTLA4Ig [24]. For all rAdVs, a green fluorescent protein (GFP) gene was inserted by Agilent Technologies to monitor the infection efficiency of the recombinant viruses. The empty rAdV with GFP (rAdV-GFP) was used as a control for the influence of viral infection on immuno-corrective therapy. All viral vectors were transfected into HEK293 cells and amplified three to five times in the same cell line. After purification, the rAdVs were titered using a plaque formation assay and stored at—80°C until use.

The rAdVs were generated, amplified, and purified according to the instructions provided by Agilent Technologies. Purified rAdVs were verified by transmission electron microscopy. The rAdVs displayed typical topological characteristics of adenoviruses, with a diameter of 80 nm. The insert DNA fragments were confirmed by DNA sequencing. To determine the optimal multiplicity of infection (MOI), rAdVs were titrated using a plaque formation assay. The expression of GFP and CTLA4Ig from the rAdVs was verified in HEK293 cells. All rAdVs showed high transduction efficacy and stable expression of the targeting proteins at an MOI of 100:1.

### DC isolation, induction, and modification

Briefly, bone marrow cells were harvested from the femurs and tibias of 6- to 8-week-old BALB/c mice on an OVA- and Derp-free diet and then were cultured in six-well plates (Life Technologies) at a density of 3×10^5^ cells per well in 2 mL RPMI-1640 medium (Life Technologies), supplemented with 100 μg/mL streptomycin (Invitrogen, Grand Island, NY), 2 mM L-glutamine (Sigma), 50 mM 2-mercaptoethanol (Sigma), and 10% fetal bovine serum (Life Technologies).

The DCs were induced by adding 20 ng/mL recombinant mouse granulocyte- macrophage colony-stimulating factor (rmGM-CSF; R&D Systems, Minneapolis, MN) and 10 ng/mL recombinant murine interleukin 4 (rmIL-4; R&D Systems) to the culture medium. The medium was changed every 2 days. Non-adherent cells were harvested after 7 days.

For the rAdVs-mediated genetic modification of DCs, 7-day cultured cells with typical DC clusters were collected and infected with rAdV-CTLA4Ig or rAdV-GFP at the optimized MOI of 100 for 2 h in serum-free medium. Next, these cells were incubated in complete medium containing rmGM-CSF and rmIL-4 for 2 days. The DCs cultured for 7 days without rAdV modification were the controls. To generate DCs that presented OVA and Derp1, all cells were cultured in the presence of 50 μg/mL whole OVA protein and 5 μg/mL Derp for 2 days. Then the cells were collected for flow cytometric analyses or for use in subsequent experiments.

### Fluorescence-activated cell sorting (FACS) analysis

CTLA4Ig, MHC-II, and the co-stimulatory molecules CD86 and CD80 expressed on the DC surface were analyzed by FACS. The cells were stained using the following primary antibodies (mAbs; eBioscience, San Diego, CA): phycoerythrin (PE)-Cy5 conjugated anti-CTLA4, PE-conjugated anti-MHC-II, PE-conjugated anti-CD80, and PE-conjugated anti-CD86. After washing with PBS, the cells were analyzed using a FACSCalibur flow cytometer (BD Biosciences). A minimum of 10^4^ events within the gated live population was collected per sample. The data were analyzed with the Cell Quest Pro analysis software by gating on the live cell populations. Appropriate isotype-matched antibody controls from the respective manufacturers were used. The infection ratio of the rAdVs-infected DCs was verified by GFP expression.

### Determination of CTLA4Ig levels in culture supernatants

The levels of CTLA4Ig in the supernatant of cultured DCs were assayed using the enzyme-linked immunosorbent assay (ELISA) (Bender, Vienna, Austria), according to the manufacturer’s instructions [23, 24].

### IgE and cytokine measurement and cell subset analysis

Blood was collected by retro-orbital bleeding and the serum was prepared and stored at—80°C. Bronchoalveolar lavage fluid (BALF) was collected by lavaging the lungs three times with 0.5 mL PBS, and then the cell suspensions were centrifuged (1500 rpm, 5 min). After centrifugation, supernatants were collected and stored at—80°C for IgE and cytokine analysis. The BALF cells were resuspended in PBS, and the total leukocyte counts were determined using a hemocytometer. Differential counts were determined by cytocentrifugation of 30 μL aliquots of BALF cells at 1500 rpm for 3 min onto slides. Next, the slides were stained with Wright-Giemsa and counted in a blinded fashion. A minimum of 200 cells were counted per sample by light microscopy.

IgE was measured using a two-site ELISA (MyBioSource, San Diego, CA). The cytokine levels in the BALF and serum were determined using a commercially available ELISA kit according to the manufacturer’s protocol. ELISA kits for IL-4 and interferon (IFN)-γ were purchased from ShiZhengBo (Beijing, China).

### Mixed lymphocyte reactions

As we previously reported [23], a one-way mixed lymphocyte reaction (MLR) assay was used. Splenic CD4+ T cells purified magnetically from C57BL/6 mice using CD4+ microbeads (Dynal; Invitrogen) served as responders. Responder cells at a density of 1×10^5^ cells/well were cultured with 2×10^3^, 5×10^3^, 1×10^4^, or 2×10^4^ DCs, or various modified DCs inactivated by 30 mg/L mitomycin C (MMC) (Roche, Indianapolis, IN, USA) in round-bottomed 96-well plates for 5 days. For the final 18 h of culture, 1 μCi [3]-HTdR was added to each well. Cultures were harvested onto glass fibers, and the thymidine incorporation was quantified using a multipurpose scintillation counter (MoSu, Shanghai, China). Results are expressed as mean count per minute (cpm) ± 1 standard deviation (SD).

### Histopathology

Bulging and bloodshot inflammatory cell infiltration and changes in bronchial tube pulmonary alveolus structure were verified by histopathology. Hematoxylin and eosin (H&E) staining was used. The lungs were removed and inflated with 4% paraformaldehyde. Then the tissues were embedded in paraffin wax and cut into sections (5 μm thick), which were stained using a standard H&E protocol.

### Airway responses

Changes in the enhanced pause (Penh) associated with aerosolized methacholine administration were used as a measure of airway reactivity. Briefly, unanesthetized mice were placed within small-volume (~600 mL) chambers equipped with transducers that monitored changes in chamber pressure as a function of each mouse’s breathing pattern (Buxco, Wilmington, NC). Penh, a calculated parameter that is a function of the degree of bronchoconstriction in response to methacholine, was calculated conventionally (BioSystem XA software, Buxco) from amplified expiratory pressure signals generated by the transducers [23]. Methacholine dissolved in PBS (pH = 7.4) was administered to the mice as an aerosol within the chambers, using a DeVilbiss ultrasonic nebulizer (aerosol droplet size = 1–5 μm), connected to an aerosol driver and pump apparatus (Buxco). Each methacholine concentration was administered for 2 min, followed by a 3-min observation period with continuous data collection. The highest Penh value achieved during the administration and observation periods was used as the peak response value for each mouse.

### Statistical analyses

The results are expressed as the mean ± standard deviation (SD). An analysis of variance (ANOVA) was used to determine the differences between all groups. Pairs of groups were compared using Student’s *t*-test. *P*-values < 0.05 were considered to indicate statistical significance.

## Results

### rAdV-CTLA4Ig-modified DCs secrete high levels of IL-10 and low levels of IL-12, and result in the low stimulation of T cells

As we previously reported [23, 24], CTLA4 was undetectable without rAdV-CTLA4Ig modification. Of rAdV-CTLA4Ig-modified DCs, most of the CTLA4Ig was secreted and reached 40–80 ng/mL in the supernatant. Compared with DCs treated with OVA alone or rAdV-GFP, rAdV-CTLA4Ig modification significantly suppressed CD80 and CD86 expression without influencing the expression of MHC-II on the surface of DCs (data not shown).

**Fig 1:**
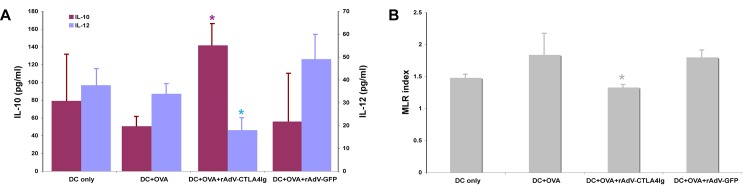
*In vitro* evaluation of the function of DCs modified by rAdV-CTLA4Ig. A, IL-10 (left Y-axis) and IL-12 (right Y-axis) secretion of modified DCs. B, characteristics of T cell stimulation of modified DCs evaluated by MLR. MLR, mixed lymphocyte reaction.

To evaluate whether CTLA4Ig could change the capacity of DCs to initiate primary immune responses, the characteristics of cytokine secretion and the ability of modified DCs to stimulate T cells were also studied *in vitro*. As shown in Fig 1A, CTLA4Ig-modified DCs secreted significantly more IL-10 than did DCs treated under different conditions. By contrast, CTLA4Ig-modified DCs secreted significantly less IL-12. The MLR tests showed that OVA and OVA+rAdV-GFP-treated DCs resulted in the stimulation of T cells. In contrast, the MLR index of CTLA4Ig-modified DCs was significantly lower than those of OVA and OVA+rAdV-GFP-treated DCs, which suggested that CTLA4Ig-modified DCs did not stimulate T cells (Fig 1B).

In summary, these data suggest that CTLA4Ig has immunosuppressive effects, whereas OVA and rAdV-GFP have immune-activating capabilities.

### Mice transplanted with OVA + rAdV-CTLA4Ig-modified DCs and sensitized with OVA in the neonatal period show immune tolerance against OVA-induced asthma at 3 months old

**Fig 2:**
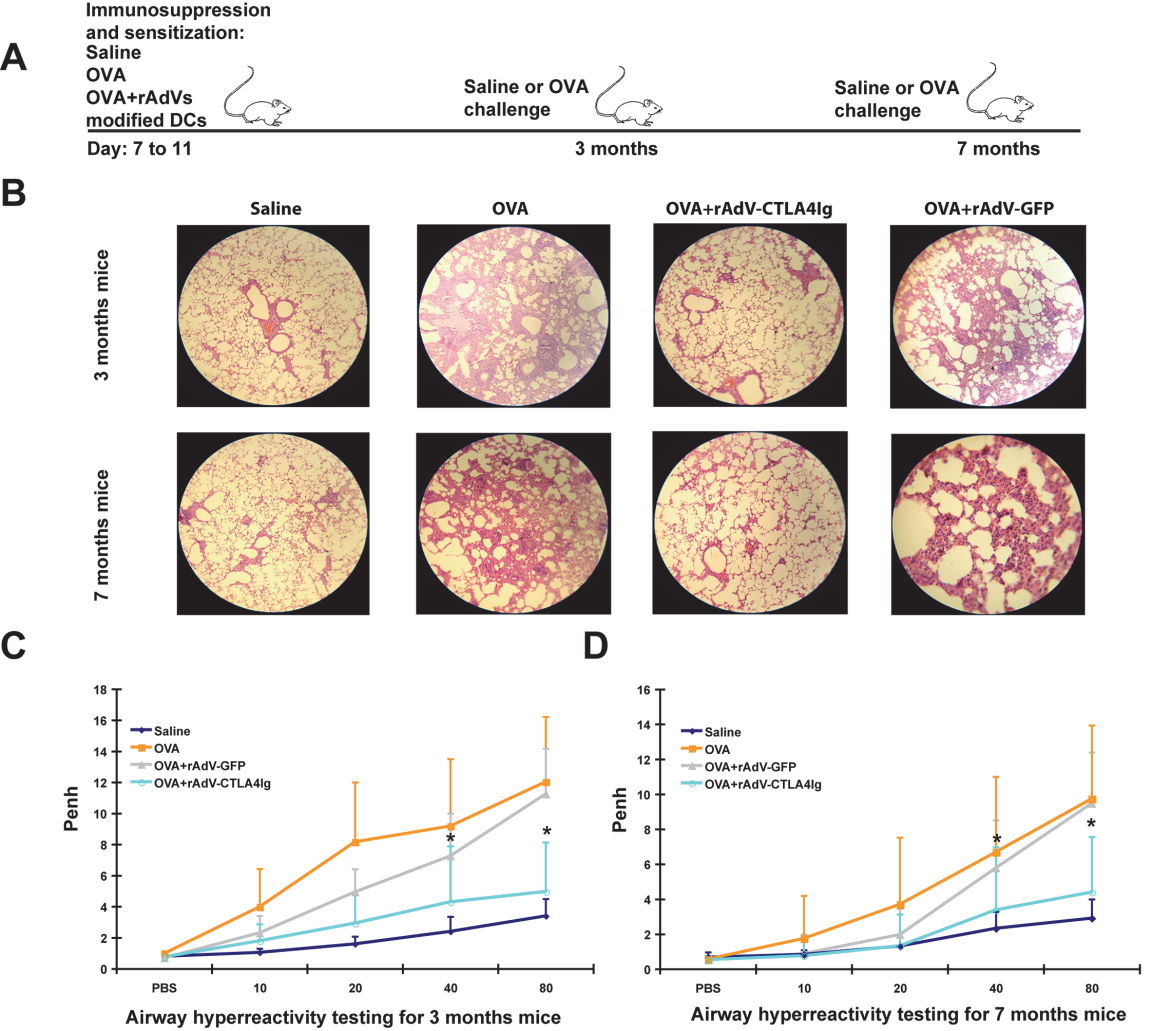
Persistence of immune tolerance to asthma antigens in the adult stage. Saline and OVA represented mice treated, sensitized and challenged by saline and OVA respectively. OVA+rAdV-CTLA4Ig and OVA+rAdV-GFP represented mice treated with OVA+rAdV-CTLA4Ig and OVA+rAdV-GFP in the neonatal period sensitized and challenged by OVA at 3 and 7 months old. Each group contains 10 mice. A, regimen of sensitization, challenge, and treatment. B, representative histopathological characteristics of mice lung: upper, lungs of 3-month-old mice; lower, lungs of 7-month-old mice. C, airway reactivity testing in 3-month-old mice. * indicates significantly low airway reactivity of OVA+rAdV-CTLA4Ig treated mice versus the asthma mice (OVA group). D. airway reactivity testing in 7-month-old mice. * indicates significantly low airway reactivity of OVA+rAdV-CTLA4Ig treated mice versus the asthma mice (OVA group).

To evaluate whether mice transplanted with OVA + rAdV-CTLA4Ig-modified DCs in the neonatal period might possess immune tolerance against asthma allergens at 3 months old, 7- to 11-day-old neonatal mice were treated with saline, OVA, OVA plus rAdV-CTLA4Ig-modified DCs, and OVA plus rAdV-GFP-modified DCs. At 3 months of age, the mice were challenged with OVA (Fig 2A).

The OVA-sensitized mice that were not treated with rAdV-CTLA4Ig-modified DCs in the neonatal period showed dysphoria or asthenia, nodding with breathing, erect hair, forelimb shrinkage, and urinary and fecal incontinence when challenged with OVA. The mice transplanted with rAdV-GFP-modified DCs in the neonatal period displayed mild asthma manifestations and pathological damage in the lung, consistent with the *in vitro* experiments described above, in which rAdV infection might activate DCs. For the mice treated with OVA+rAdV-TCLA4Ig-modified DCs, all of the asthma symptoms mentioned above were greatly improved, and the respiratory rate decreased to 115±11 beats per minute, similar to normal controls. The lungs of asthmatic mice were bulging and bloodshot, with inflammatory cell infiltration and bronchial tube pulmonary alveolus structural damage (Fig 2B, upper). Of the mice transplanted with OVA+rAdV-TCLA4Ig-modified DCs, the pathological damage in the lungs was lower (Fig 2B, upper).

To observe changes in cellular and humoral immunity in all mouse groups, both the BALF and sera of the mice were subjected to analyses of cellular composition and cytokine levels. In BALF, the levels of total white blood cells and eosinophils in the asthma group were 176.12±17.51 and 62.25±6.67×10^4^/mL respectively, significantly higher than in healthy control mice (p < 0.05; Table 1, left). The IgE and IL-4 levels of the asthma group were significantly higher than those of the control mouse group, and the IL-10 and IFN-γ levels of asthma group were significantly lower than those of the healthy controls (p < 0.05), whereas the IFN-γ levels among non-control groups were similar, suggesting that CTLA4Ig treatment did not change the IFN-γ level in BALF (Table 1, left). These indices were similar in the mice infected with rAdV-GFP DCs, suggesting that rAdV-GFP caused changes in cellular and humoral immunity (Table 1, left). The levels of white blood cells and eosinophils in mice treated with OVA+rAdV-CTLA4Ig DCs were 87.25±8.08 and 18.50±1.85×10^4^/mL, respectively, significantly lower than the asthma and OVA+rAdV-GFP DCs groups (p < 0.05). The levels of IgE and IL-4 of the OVA+rAdV-CTLA4Ig DCs mice were significantly lower than those of asthmatic mice, and the IL-10 level was significantly higher than that of the asthmatic mice but lower than that of the control mice (Table 1, left). In sera, the levels of IgE and IL-4 were significantly higher in asthma mice than in control mice; the levels of IFN-γ were significantly lower in all treatment groups than in control mice; and the levels of IL-10 were significantly higher in the OVA+rAdV-CTLA4Ig DCs mice than in the other groups. The values for the OVA+rAdV-GFP DCs group were similar to those of the asthma group (Table 1, left). The levels of IgE and IL-4 were significantly higher in the OVA+rAdV-CTLA4Ig DCs mice than in controls, but significantly lower than in asthmatic mice (Table 1, left).

**Table 1:** Cell classification and changes in cytokine levels 3 and 7 months after sensitization.

		Mice at 3 months			Mice at 7 months		
	Control	Asthma	OVA+rAdV-CTLA4Ig	OVA+rAdV-GFP	Asthma	OVA+rAdV-CTLA4Ig	OVA+rAdV-GFP
*BALF*							
Total white blood cells (x10^4^/ml)	28.50±4.10	176.12±17.51*	87.25±8.08* ^▲^	170.00±14.42	/	/	/
Eosinophilic cells (x10^4^/ml)	0.75±0.70	62.25±6.67*	18.50±1.85* ^▲^	59.13±6.10	69.13±5.00*	14.03±4.31* ^▲^	65.00±4.63
IgE (10^3^ pg/ml)	5.49±1.91	122.04±59.58*	22.85±11.81* ^▲^	101.19±35.31	85.86±16.18*	27.90±9.42* ^▲^	84.27±19.95
IL-4 (pg/ml)	32.04±0.30	73.29±8.69*	46.91±2.90* ^▲^	79.15±4.91	58.23±12.10*	37.91±11.00* ^▲^	55.14±10.80
IL-10 (pg/ml)	225.70±10.19	27.07±11.37*	83.20±42.40* ^▲^	31.70±7.37	23.59±5.80*	59.13±10.68* ^▲^	21.98±5.16
IFN gamma (pg/ml)	121.53±13.93	78.63±2.78*	62.30±0.94*	70.14±1.37	51.41+11.27*	56.34+11.83* ^▲^	54.01+12.32
*Sera*							
IgE (10^5^ pg/ml)	1.86±0.65	5.30±1.73*	3.93±1.28* ^▲^	5.32±1.76	5.06±0.41*	3.91±0.28* ^▲^	5.20±0.40
IL-4 (pg/ml)	29.84±14.66	88.67±9.17*	70.52±6.95* ^▲^	89.83±7.92	51.69±11.45*	30.21±14.60* ^▲^	49.74±9.21
IL-10 (pg/ml)	320.58±9.40	226.83±10.64*	441.95±8.17* ^▲^	321.82±9.17	221.55±26.16*	422.26±26.16* ^▲^	218.81±21.94
IFN gamma (pg/ml)	132.47±12.35	78.71±5.83*	76.38±6.28*	70.72±2.68	74.35±10.41*	82.79±12.27* ^▲^	73.81±10.11

BALF, bronchoalveolar lavage fluid; IL, interleukin; IFN, interferon; CTLA4Ig, cytotoxic T lymphocyte antigen 4-immunoglobulin; GFP, green fluorescent protein; IgE, immunoglobulin E. The mice sensitized and challenged by saline were used as controls; the mice sensitized and challenged by OVA were defined as the asthma group; the mice transplanted with DCs modified by treatment with OVA+rAdV-CTLA4lg before being sensitized to OVA were defined as OVA+rAdV-CTLA4Ig; the mice transplanted with DCs modified by treatment with OVA+rAdV-GFP before being sensitized to OVA were difined as OVA+rAdV-GFP.

*, P<0.05 compared to controls;

▲, P<0.05 compared to the asthma group.

The airway reactivity of mice that were not treated with OVA+rAdV-CTLA4Ig-modified DCs in the neonatal period increased as the concentration of methacholine increased. OVA+rAdV-GFP treatment did not improve airway reactivity. The airway reactivity of the mice treated with OVA+rAdV-CTLA4Ig displayed little change as the concentration of methacholine increased, and showed significantly low reactivity at methacholine concentrations of 40 and 80 mg/mL compared to asthmatic mice (Fig 2C). These findings indicate that mice treated with OVA+rAdV-CTLA4Ig-modified DCs in the neonatal period show tolerance to OVA-induced asthma as adults (3 months old).

### Persistence of the therapeutic effects

Does this immune-tolerant capacity decrease with growth? To evaluate this, persistence was further studied in 7-month-old mice. As shown in Fig 2D, the airway reactivity of mice without DC transplantation in the neonatal period increased as the concentration of methacholine increased, indicating that the mice could be sensitized and asthma could be induced by OVA. The OVA+rAdV-GFP treatment did *not* improve airway reactivity. The airway reactivity of the mice that did receive DC transplantation in the neonatal period displayed slight increases as the concentration of methacholine increased, and showed significantly low airway reactivity at methacholine concentrations of 40 and 80 mg/mL versus the asthma mice. These mice also showed similar lung tissue pathology, changes in cellular and humoral immunity in BALF samples, and changes in humoral immunity in sera samples as mice observed at 3 months, indicating significant immune tolerance at 7 months of age against asthma induced by OVA (Table 1, right and Fig 2B, bottom).

### Specificity of the therapeutic effects

**Fig 3:**
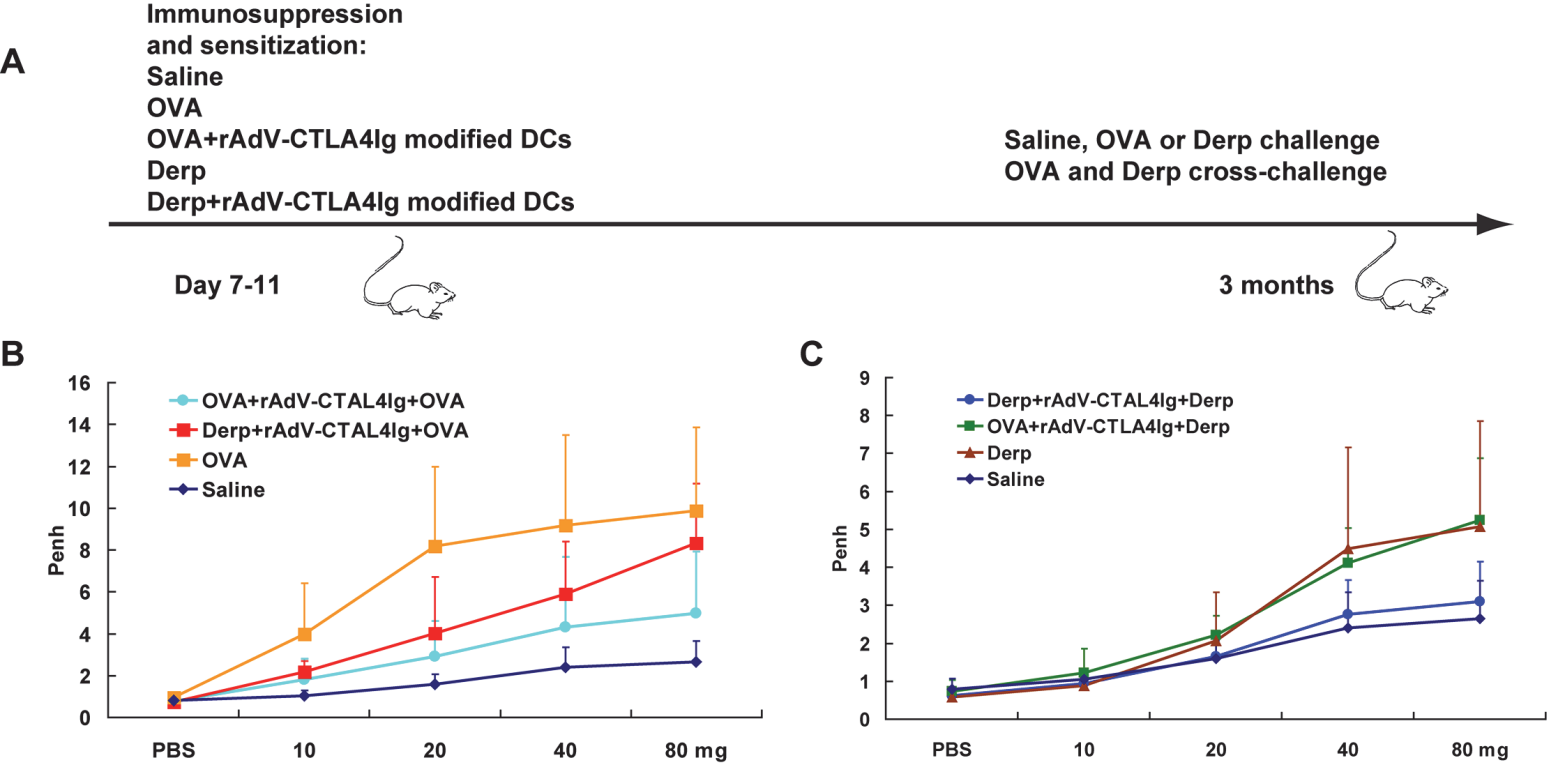
Specificity assessment. To evaluate whether the persistent immune tolerance was allergen-specific, the neonatal mice were treated with OVA+rAdV-CTLA4Ig-modified DCs or Derp+rAdV-CTLA4Ig-modified DCs, 3 months later, the specificity of the therapeutic strategy was assessed by allergen cross challenge. Each group contains 10 mice. Saline, OVA and Derp represented mice treated, sensitized and challenged by saline, OVA and Derp respectively. OVA+rAdV-CTLA4Ig+OVA represented mice treated with rAdV-CTLA4Ig, sensitized by OVA and challenged by OVA. Derp+rAdV-CTLA4Ig+OVA represented mice treated with rAdV-CTLA4Ig, sensitized by Derp and challenged by OVA. Derp+rAdV-CTLA4Ig+Derp represented mice treated with rAdV-CTLA4Ig, sensitized by Derp and challenged by Derp. OVA+rAdV-CTLA4Ig+Derp represented mice treated with rAdV-CTLA4Ig, sensitized by OVA and challenged by Derp. A, the regimen of sensitization, challenge, and treatment in specificity assessment. B, airway reactivity testing of OVA-sensitized mice challenged by OVA or Derp. C, airway reactivity testing of Derp-sensitized mice challenged with Derp or OVA.

To evaluate whether the persistent immune tolerance was allergen-specific, the neonatal mice (7–11 days old) were treated with OVA+rAdV-CTLA4Ig-modified DCs and Derp+rAdV-CTLA4Ig-modified DCs. Then, 3 months later, the specificity of the therapeutic strategy was assessed by allergen cross challenge (Fig 3A).

For airway reactivity, the mice treated with OVA+rAdV-CTLA4Ig-modified DCs in the neonatal period displayed higher reactivity when challenged with Derp and lower reactivity when challenged with OVA (Fig 3B). The mice treated with Derp+rAdV-CTLA4Ig-modified DCs showed the same trend (higher reactivity to OVA, lower reactivity to Derp; Fig 3C).

Regarding histopathology, transplantation of antigens plus rAdV-CTLA4Ig-modified DCs in the neonatal period displayed a protective effect against the same allergens that induced lung histopathological damage (data not shown).

**Fig 4:**
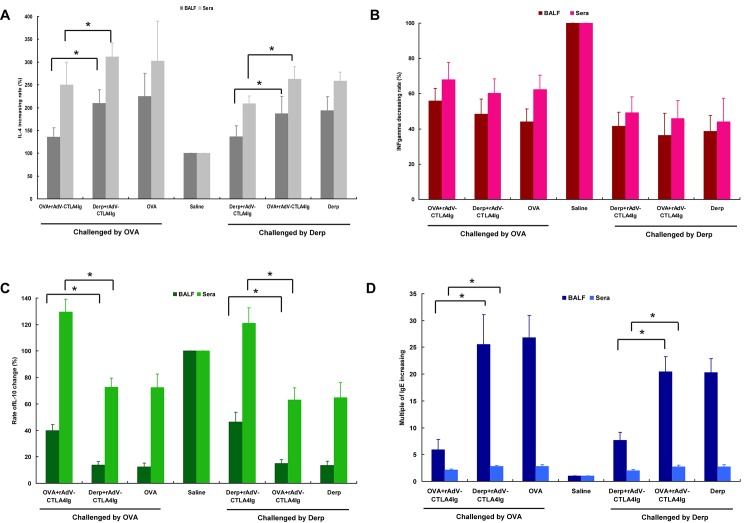
Humoral immunological changes in the specificity assessment. For easy comparisons, the rates of change in IL-4, IL-10, IFN-γ, and IgE in both BALF and sera were adjusted by the values of the control (sensitized and challenged by saline). * indicates statistical significance of each comparison. A, the increasing rates of IL-4 in the regimens of sensitization, challenge, and treatment. B, IFN-γ decreasing rates in the regimens of sensitization, challenge, and treatment. C, IL-10 changes in the regimens of sensitization, challenge, and treatment. D, the increasing rates of IgE in the regimens of sensitization, challenge, and treatment.

Regarding humoral immunological changes, all mice that received transplantation of DCs treated with OVA, OVA+rAdV-CTLA4Ig, or Derp+rAdV-CTLA4Ig in the neonatal period had increased levels of IL-4 and IgE in both BALF and sera, although treatment with CTLA4Ig significantly slowed their respective rates of increase in mice sensitized to and challenged with OVA but not Derp (Fig 4A and 4D). The IFN-γ levels of all mice transplanted with DCs in the neonatal period showed significant declines compared to saline-treated mice, but there were no significant differences among the treatment groups (Fig 4B). IL-10 levels in BALF decreased significantly in mice transplanted with DCs treated with OVA, OVA+rAdV-CTLA4Ig, and Derp+rAdV-CTLA4Ig, although treatment with CTLA4Ig significantly slowed their respective rates of decrease in mice sensitized to and challenged with OVA but not Derp (Fig 4C). In sera samples, although the levels of IL-10 decreased in mice transplanted with DCs treated with OVA and Derp+rAdV-CTLA4Ig, the levels significantly increased in mice transplanted with DCs treated with OVA+rAdV-CTLA4Ig (Fig 4C).

Of the mice challenged with Derp, similar results were observed. That is, humoral immunological changes showed that CTLA4Ig induced allergen-specific immune tolerance (Fig 4).

## Discussion

We explored a new therapeutic strategy for asthma in mouse models. Mice transplanted with DCs modified by rAdV-CTLA4Ig and allergens before allergen sensitization in the neonatal period displayed satisfactory protective effects against asthma at the 3-month-old stage when challenged with allergens. The immune tolerance was maintained at least up to 7 months old, which is longer than current ASIT; the latter is based on the principle of *peripheral* immune tolerance, which is focused on allergen-specific T cell suppression. Our study suggests that early immune interference via DCs is a promising strategy for asthma prevention.

The CD80/CD86-CD28 axis is a critical target for immuno-corrective therapy because CD28, engaged by CD80 or CD86, concomitant with TCR signaling, is sufficient to fully activate a resting naïve T cell. Conversely, inhibition of the CD80/CD86-CD28 axis prevents T-cell activation *in vitro* and *in vivo* [21]. Studies have shown that CD28 is essential for the development of allergic airway inflammation in a number of preclinical models [25–27]. CTLA4 has one log higher competitive binding activity to CD80/CD86 than CD28 and is widely used in immuno-corrective therapy [22]. Numerous studies have demonstrated that treatment with various species of CTLA4Ig can affect several diseases, such as preventing contact hypersensitivity [28, 29], acquired immune deficiency syndrome [30, 31], psoriasis vulgaris [32], and asthma [23, 24].

*In vitro* experiments have shown that DCs genetically engineered with CTLA4Ig show immunosuppressive activity, characterized mainly by increased IL-10 secretion and decreased IL-12 secretion and the stimulation of T cells. DCs genetically engineered to express IL-10 have been reported to induce long-lasting antigen-specific tolerance in experimental asthma models [33]. Allergic airway diseases are associated with skewed Th2 cytokine production [23], although the underlying cause of this aberrant immune response is not well understood. IL-12 may play pivotal roles in the Th2-polarized immune response to inhaled allergens [34]. IL-12 is a critical determinant of Th1-mediated immune responses, and deficiency in this cytokine can lead to Th2-polarized immune responses [34]. Impaired IL-12 production is always observed in asthmatic individuals [35]. Our *in vitro* data are consistent with the above reports and indicate a possible mechanism of *in vitro* CTLA4Ig-modified DC transplantation therapy.

Humoral immunity assessment showed that both OVA and Derp increased IL-4 and IgE levels, in both BALF and sera in the mouse model. In addition, CTLA4Ig-modified DCs displayed significant inhibiting effects on production and secretion. The IFN-γ levels of all mice groups showed a decline of about half compared to saline-treated mice. This might suggest that CTLA4Ig-modified DCs improve asthma, but not via Th1 cells, although we are not sure why OVA, OVA+rAdV-CTLA4Ig, and OVA+rAdV-GFP-modified DC transplantation induced similar IFN-γ changes in mice. Another interesting result is that both OVA and Derp may induce declines in IL-10 levels in BALF and CTLA4Ig might slow this decrease, but the mice treated with CTLA44Ig-modified DCs showed significantly higher IL-10 levels in sera. This phenomenon might partially support a possible explanation that this immune-tolerant strategy might stimulate IL-10 production and control the distribution of IL-10 in the lung simultaneously. Although we did not conduct analyses on Th1 and Th2 cells directly, our humoral immunity assessment showed that the levels of IL-4, a Th2 cytokine, in CTLA4Ig-modified DC-treated mice increased significantly compared to asthmatic mice, suggesting functional changes in Th1.

Central tolerance refers to self-tolerance that is achieved at the level of the central lymphoid organs. Developing B and T cells in the bone marrow and thymus, which recognize self-antigen, face deletion or marked suppression [36, 37]. In the present study, although we observed that immunosuppression in the early postnatal period induced a persistent, allergen-specific immune tolerance to asthma in adult mice, our data did not clarify whether the reduced response to allergen upon challenge is due to the deletion of allergen-specific immune cells in the central lymphoid organs, or the suppression of allergen-specific immune cells by the peripheral tolerance mechanism. Our data showed that although the immune response was significantly suppressed in the OVA+rAdV-CTLA4Ig or Derp+rAdV-CTLA4Ig groups compared to each asthma control group when challenged with the corresponding allergens, the cellular and humoral immunity detection indexes showed that the immune response of a certain intensity did occur in the OVA+rAdV-CTLA4Ig or Derp+rAdV-CTLA4Ig groups when compared with the saline controls. These phenomena suggest that allergen-specific immune responses are suppressed rather than deleted.

Since asthma is highly prevalent and seriously affects the quality of life of patients, and current clinical treatments of asthma are limited, there is a continuing need for effective therapeutic strategies to advance the clinical treatment of asthma. Our study provides a persistent immune tolerance strategy for asthma prevention. This strategy may be understood as a new concept in vaccines. Specifically, we could identify the specific asthma allergens, orient the DCs with these allergens and CTLA4Ig, and then transplant these modified DCs into a newborn showing susceptibility to asthma. Our data show that this persistent immune tolerance strategy is highly allergen-specific and does not alter the overall function of the host immune system, and thus the strategy seems to be very safe.
